# Epithelial Cell–Specific Prognostic Signature (FTH1, RIT1, WASL, NDRG2, KIFC3) Stratifies Cervical Cancer Patients and Correlates With Immune Infiltration

**DOI:** 10.1155/humu/4109928

**Published:** 2026-02-06

**Authors:** Xuegu Wang, Xingchen Pan, Xiang Li, Biao Ding, Zhixin Jin, Xiaojing Wang, Chengli Dou

**Affiliations:** ^1^ Department of Obstetrics and Gynecology (Center for Reproductive Medicine), The First Affiliated Hospital of Bengbu Medical University, Bengbu, China, bbmc.edu.cn; ^2^ School of Life Sciences, Bengbu Medical College, Bengbu, China, bbmc.edu.cn; ^3^ Molecular Diagnostic Center, The First Affiliated Hospital of Bengbu Medical University, Bengbu, China, bbmc.edu.cn; ^4^ Anhui Province Key Laboratory of Clinical and Preclinical Research in Respiratory Disease, Bengbu, China

**Keywords:** cervical cancer, computational analyses, epithelial cell, prognostic biomarker, Riskscore model

## Abstract

**Background:**

Cervical cancer (CC) remains one of the leading female malignancies. Epithelial cells (EpCs), primarily derived from the cervical squamous and glandular epithelium, are targeted by human papillomavirus to drive CC. Herein, we aimed to develop an EpC‐specific risk model to improve clinical outcomes and unravel tumor immune microenvironment alterations in CC.

**Methods:**

scRNA‐seq data from GSE208653 were processed using Seurat (including SCTransform for normalization and Harmony for batch correction). EpC heterogeneity was analyzed via subclustering, pseudotime trajectory analysis with monocle2, and cell–cell communication inference with CellChat. The hdWGCNA package identified EpC‐specific coexpression modules. Prognostic genes were screened by univariate Cox and LASSO regression, and a Riskscore model was built using multivariate Cox regression. Immune infiltration was assessed by ssGSEA, MCPCounter, and ESTIMATE algorithms. Drug sensitivity correlation was analyzed using pRRophetic. In vitro functional assays validated key gene roles in CC cells.

**Results:**

Forty thousand four hundred fifty‐seven cells were annotated into eight cell populations with a lower percentage of EpCs. Thereafter, EpCs were categorized into three subclusters with specifically highly expressed genes in peculiar biological pathways and with distinct trajectories of fate. A strong cell–cell communication network was observed, particularly involving Ep C3 and immune cells, via ligand–receptor pairs such as LGALS9‐CD44 and HBEGF‐EGFR. The hdWGCNA analysis revealed Ep C3–specific gene modules, from which a five‐gene prognostic signature (*FTH1*, *RIT1*, *WASL*, *NDRG2*, and *KIFC3*) was constructed. The resulting risk model effectively stratified patients into high‐ and low‐risk groups with significantly different overall survival in both TCGA‐CESC and GSE52903 cohorts, supported by time‐dependent ROC curves. The high‐risk group exhibited lower immune/stromal scores and distinct immune cell infiltration patterns. The risk score significantly correlated with sensitivity to several chemotherapeutic agents. Crucially, in vitro experiments confirmed that *FTH1* knockdown inhibited the proliferation, migration, and invasion of CC cells while enhancing the level of apoptosis in cancer cells.

**Conclusion:**

A proposed EpC‐specific gene signature for CC may be applicable to support clinical decision‐making.

## 1. Introduction

Cervical cancer (CC) remains a major health issue worldwide, which ranks fourth in the most prevalent female cancer [1–3]. Persistent infection of human papillomavirus (HPV) is the central cause of CC [4, 5]. While CC is largely preventable through primary prevention (HPV vaccination), secondary prevention (cervical screening and treatment of precancerous lesions), and tertiary prevention (early diagnosis and treatment), such effective prevention remains suboptimal in some resource‐constrained settings [6]. Current treatment strategies for CC patients encompass surgery, radiotherapy, and conventional chemotherapy, with the recent integration of immunotherapy (e.g., PD‐1/PD‐L1 inhibitors) and targeted therapies (e.g., antiangiogenic agents like bevacizumab) showing promising results in advanced or recurrent cases [7]. However, treatment efficacy varies significantly among patients, highlighting the urgent need for better prognostic biomarkers and personalized therapeutic approaches. Although HPV vaccination is estimated to reduce the incidence of HPV‐associated CC, achieving its complete elimination will require time, underscoring the continued importance of understanding the detailed molecular mechanisms underlying CC progression to improve patient management [8].

Currently, available gene expression profile data and associated bioinformatics analyses have emerged as a promising novel research direction, as exemplified by studies demonstrating bioinformatics applications in efficiently organizing and analyzing biological data and experimental results in bioscience [9–11]. Recent evidence supports single‐cell RNA sequencing (scRNA‐seq) for dissecting CC ecosystem heterogeneity, as it identifies complex cell populations and probes tumor microenvironment (TME) molecular heterogeneity at single‐cell resolution [12, 13]. Accumulating evidence has laid great emphasis on the role of epithelial cells (EpCs) in tumor initiation, progression, and metastasis of CC [14]. HPV is known to infect EpCs, with its replication cycle tightly linked to epithelial differentiation [15]. This long‐term infection drives EpCs to invasive cancer via the accumulation of DNA alterations in host oncogenes and tumor suppressor genes, including epigenetic and genetic changes [16]. Moreover, while examining the application of scRNA‐seq in CC, a prior exploration has already unveiled the intra‐ and intertumoral heterogeneity of EpCs in HPV^+^ cervical adenocarcinoma (CAde) [17]. Such evidence, accordingly, made us curious to further explore the heterogeneity of EpCs in the context of CC so as to fathom out some relevant molecular mechanisms driving the progression of CC.

In our current study, therefore, the data of scRNA‐seq and bulk RNA sequencing (RNA‐seq) were incorporated to reveal the heterogeneity of EpCs in CC, and then the feature genes related to EpCs were unraveled using high‐dimensional weighted gene coexpression network analysis (hdWGCNA). Thereby, a relevant risk model was established, and the immune infiltration and drug sensitivity were further explored. It is expected that these results would provide novel insights into the role of EpCs in CC and some preliminary reference for the personalized therapy of CC.

## 2. Methods

### 2.1. Data Source and Preprocessing

Bulk RNA‐seq data: The RNA‐seq data containing the gene expression data of patients were downloaded from UCSC Xena and hereafter referred to as the cohort The Cancer Genome Atlas (TCGA)—cervical squamous cell carcinoma (CSCC) and endocervical adenocarcinoma (CESC). Thereafter, the samples with complete clinical follow‐up info and survival > 30 days were retained, and the Ensembl was then converted to gene symbol. The maximum value was taken when there were multiple gene symbols. Finally, a total of 273 primary tumor samples were obtained. Also, the RNA‐seq data and clinical information of the dataset GSE52903 were downloaded from Gene Expression Omnibus (GEO), the probes were converted to symbols, and the samples with survival > 30 days were obtained. Fifty‐four samples were then collected [18].

scRNA‐seq data: The two normal samples and three HPV‐infected CC samples of the dataset GSE208653 were applied for the scRNA‐seq analysis [19]. For the filtering of the data, the cells with the criteria were retained, including each gene expressed in at least three cells and each cell expressing more than 100 genes [20]. Then, the cells were further filtered based on the three criteria: (1) nFeature_RNA > 300, (2) nCount_RNA ≤ 100,000, and (3) percent.mt < 15%. Following the standardization using the SCTransform function and the principal component analysis (PCA) via the RunPCA function, the intersample batch effects were removed via the Harmony package [21]. Cell populations were thereafter clustered using the functions FindNeighbors and FindClusters, and the uniform manifold approximation and reduction (UMAP) dimensionality reduction was carried out in the first 30 principal components. Cell populations were further annotated using the marker genes from the CellMarker 2.0 database. The corresponding results following the quality control are shown in Figure S1.

### 2.2. Construction of the Single‐Cell Pseudotime Trajectory

The count data of EpCs were read via the monocle2 package, and the phenotype information was merged beforehand [22]. The cds object was then created using the function newCellDataSet, and the genes expressed in ≥ 10 cells were retained. Those genes differentially expressed in normal and CC samples were additionally determined with the differentialGeneTest function, followed by the dimensionality reduction via the reduceDimension function (max_components = 2 and method = “DDRTree”) and the ordering of cells via the orderCells function to plot the trajectory. The starting point of the trajectory was defined as the branches in the cells of the normal samples.

### 2.3. Functional Enrichment Analysis

The genes specifically highly expressed in EpCs and differentially expressed based on the branch points were subjected to the Kyoto Encyclopedia of Genes and Genomes (KEGG) enrichment analysis using the clusterProfiler package [23].

### 2.4. Cell–Cell Communication Analysis

The cell–cell communication status of CC and normal samples was explored with the CellChat package, and the corresponding number of interactions was visualized based on the circle plots [24]. For the communication analysis, the createCellChat function was applied to create the objects, and the overexpressed ligand–receptor pairs were identified using the identifyOverExpressedGenes and identifyOverExepressedInteractions functions. Then, the possibility of the interaction was inferred using the computeCommunProb function, and the corresponding results were visualized in the bubble plots.

### 2.5. The hdWGCNA on Sorting EpC‐Relevant Gene Modules

The rds data of single‐cell transcriptomics were read using the hdWGCNA package, and the coexpression network was constructed using 5% genes expressed in Subcluster 3 of EpCs (hereafter referred to as Ep C3) using the optimal soft threshold based on the TestSoftPowers function. The gene modules (*n* = 7, M1–M7) were then obtained, and their correlation with Ep C3 was further examined. The key genes from the feature gene modules were additionally determined following the calculation of the connectivity [25].

### 2.6. Construction and Validation of the Riskscore Model

The Top 50 genes from the feature gene modules of hdWGCNA analysis were intersected as the module genes of Ep C3, which were then subjected to univariate Cox regression analysis to obtain the prognostically relevant genes (*p* < 0.05). The least absolute shrinkage and selection operator (LASSO) and stepwise regression analyses were both applied to narrow down the gene number, and the remaining key genes were applied for the construction of the risk model based on the following formula: Riskscore = *Σ* *β* *i* × Exp*i* (*β* refers to the Cox regression coefficient and Exp denotes the gene expression level).

In the meantime, the expressions of the key genes in the samples were quantified, and the results were visualized in a heatmap [26]. The overall survival of patients (in the cohorts of TCGA‐CESC and GSE52903) with diverse risk types (which were stratified based on the optimal Riskscore value) was plotted using Kaplan–Meier curves, and the efficacy of the risk model on predicting the 1‐ to 5‐year overall survival was further examined using receiver operator characteristic (ROC) curve and the corresponding calculated area under the curve (AUC) values based on the timeROC package [27].

### 2.7. Immune Infiltration and Drug Sensitivity Analyses

For the immune infiltration analysis, the enrichment score of 28 types of immune cells in samples of diverse risk types was calculated using single‐sample gene set enrichment analysis (ssGSEA) and the MCPCounter algorithm [28]. Also, the ESTIMATE algorithm was applied to compute the following scores in patients of diverse risk types, including StromalScore, ImmuneScore, and ESTIMATEScore.

For the drug sensitivity analysis, the half‐maximal inhibitory concentration (IC_50_) of the drugs was calculated, and their correlation with the Riskscore was determined. Those with statistical significance were deemed when the *p* value was lower than 0.05 and the absolute value of the correlation (|cor|) was more than 0.3.

### 2.8. Molecular Validation on the Involvement of the Key Genes in CC

Cell culture and transfection: Human endometrial epithelial cells (hEECs, Product Co. CP‐H058) were ordered from Procell (Wuhan, China), and CC cells HeLa (Product Co. C5073) and MS751 (Product Co. C5517) were purchased from BD Bio (Hangzhou, China). All cells were cultured in Dulbecco′s modified Eagle′s medium (DMEM, PM150270, Procell, China) supplemented with 10% fetal bovine serum (FBS, F814‐500, BD Bio, China) and incubated in an incubator at 37°C with 5% CO_2_. The small interfering RNAs against *FTH1* and the scramble control were all ordered from GenePharma (Shanghai, China) and then transfected into CC cells using Lipo6000 transfection reagent (C0526, Beyotime, China) as recommended by the producer. All sequences used for the transfection are available in Table S1.

Quantification test: The quantitative real‐time PCR was applied for the quantification of the relative mRNA levels in cells. Toward this end, the total cellular RNA was extracted using an RNA extractor kit (R0011, Beyotime, China), and the concentration was accordingly quantified. Following the synthesis of the complementary DNA (cDNA) by a first‐strand cDNA synthesis kit (D7190S, Beyotime, China), the PCR assay was performed using the SYBR Green qPCR Mix (D7260, Beyotime, China) and CFX96 touch real‐time PCR system (Bio‐Rad, Hercules, California, United States). The relative mRNA expression levels were finally calculated using the method 2^−*ΔΔ*ct^ and normalized to the housekeeping control GAPDH [10]. The primers applied in this study are all shown in Table S2.

Cell viability, migration, and invasion test: For the cell viability test, the transfected CC cells HeLa and MS751 were incubated in the 96‐well plates at a density of 2 × 10^3^ cells per well for the indicated times (0, 24, 48, and 72 h). Subsequently, the cell counting kit‐8 (CCK‐8) solution from the assay kit (C0039, Beyotime, China) was added to these cells for an additional 4‐h culture. Following the culture, the optical density at 450 nm (OD_450_) was finally recorded in the microplate reader (iMark, Bio‐Rad, United States) to calculate the viability of cells in each group.

For the cell migration test, CC cells at a density of 5 × 10^5^ were cultured in 6‐well plates until growing fully confluent, following which the monolayers of cells were scratched with a sterile pipette tip. Then, the cells were washed in phosphate‐buffered saline to remove the debris and continued to be cultured for 48 h. The scratch was thereafter photographed under an inverted optical microscope (ECLIPSE Ti2‐A, Nikon Instruments Inc., Tokyo, Japan) to quantify the wound closure degree.

For the cell invasion test, the Transwell assay was implemented using the 24‐well Transwell plates (pore: 8 *μ*m, 3422, Corning Inc., Corning, New York, United States) coated with the Matrigel matrix (C0372, Beyotime, China). CC cells (8 × 10^3^) were counted and suspended in the upper chamber with the nonserum culture media (200 *μ*L), and 600 *μ*L complete medium containing 10% FBS was added to the corresponding lower compartment. After 48 h, the invading cells in the lower chamber were fixed in 4% fixative (P0099, Beyotime, China) for 30 min and stained with 0.1% crystal violet staining solution (C0121, Beyotime, China) at room temperature for 30 min. An inverted optical microscope (ECLIPSE Ti2‐A, Nikon Instruments Inc., Japan) was adopted to observe three randomly picked areas, and the number of invading cells was accordingly calculated.

### 2.9. Statistical Analyses

All computational analyses and the corresponding data visualization were all accomplished using R software (Version 3.6.3). The data of two continuous variables were compared with the Wilcoxon rank‐sum test, and the overall survival of the patients in different groups was compared based on the log‐rank test.

All laboratory analyses and the corresponding data were analyzed in GraphPad Prism software (Version 8.0.2). Unpaired *t*‐test and one‐/two‐way analysis of variance were both applied to compare the data and determine the statistical significance. Overall, the data were deemed to be statistically significant when the *p* value was below 0.05.

## 3. Results

### 3.1. Single‐Cell Landscape in CC Based on the Dataset GSE208653

The dataset GSE208653 was applied for the scRNA‐seq analysis based on the two normal samples and three HPV‐infected CC samples. Following the processes of filtering, standardization, and dimensionality, the remaining cells in the total number of 40,457 were allocated into 15 main clusters (Figure 1a). These cells were thereafter annotated as mast cell, myeloid cell, neutrophil, T cell, fibroblast, EpC, B cell, and endothelial cell (Figure 1b,c). The percentage of these annotated cell populations is further quantified and displayed in Figure 1d, where an evidently higher percentage of T cell and myeloid cell and a lower percentage of EpC were noticed.

Figure 1Single‐cell landscape in cervical cancer based on the dataset GSE208653. (a, b) UMAP plot displaying cell populations based on the dataset GSE208653 (a) before and (b) after annotation. (c) The specifically highly expressed genes in each identified cell population. The *x*‐axis represents the identified cell population, and the *y*‐axis represents the corresponding marker genes from the CellMarker 2.0 database. (d) Percentage of each identified cell population in two normal samples (GSM6360680 and GSM6360681) and three HPV‐positive samples (GSM6360686, GSM6360687, and GSM6360688) based on the dataset GSE208653. The *x*‐axis indicates the percentage of cell counts, and the *y*‐axis indicates the sample.

**Figure:**
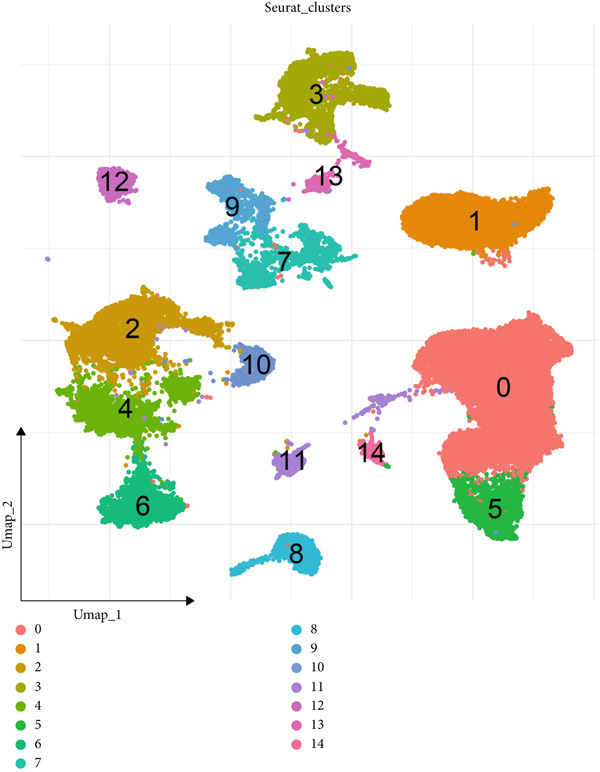
(a)

**Figure:**
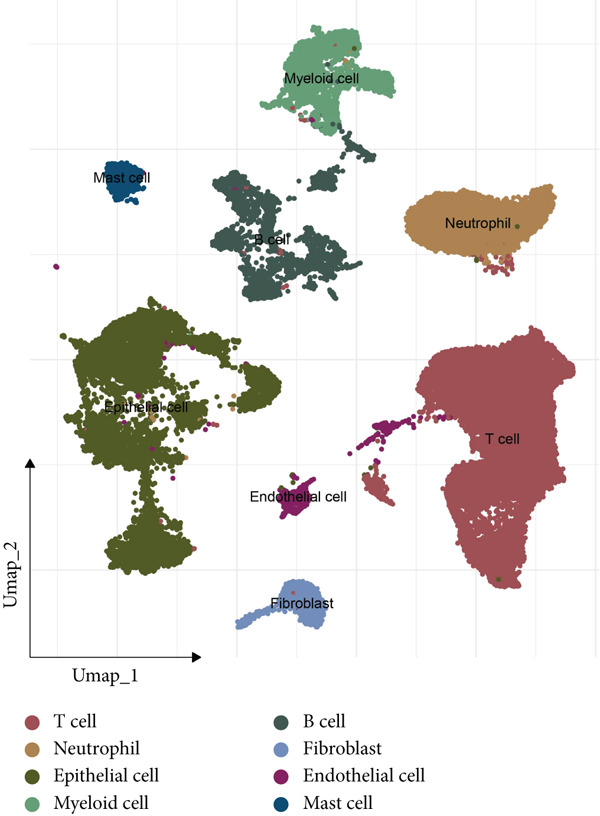
(b)

**Figure:**
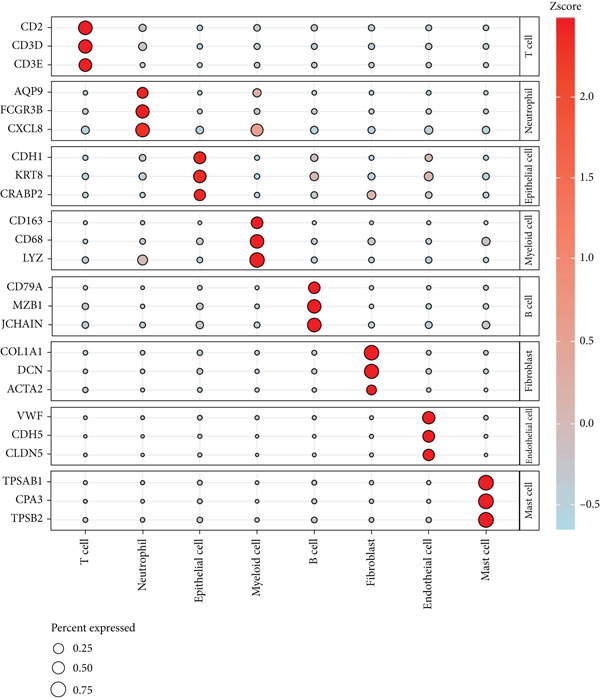
(c)

**Figure:**
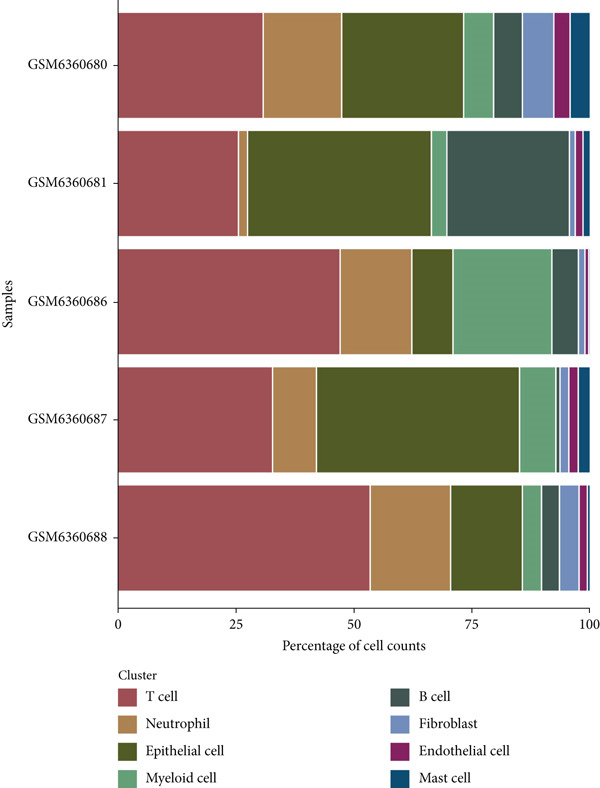
(d)

### 3.2. Heterogeneity of EpCs in CC Based on the Dataset GSE208653

Combining the conclusion from an existing study highlighting the role of EpCs in CAde [17], we thereafter aimed to fathom out the specific role of EpCs in CC, then the t‐SNE clustering on the EpCs was performed, and EpCs were accordingly allocated into three main subclusters with their specifically highly expressed genes (Ep C1–Ep C3; Figure 2a,b). Then, the specifically highly expressed genes in the three subclusters of EpCs were subjected to the KEGG enrichment analysis (Figures 2c, 2d, and 2e). The corresponding data have suggested that genes in the Ep C1 subcluster were enriched in ribosome, spliceosome, and oxidative phosphorylation, genes in the Ep C2 subcluster were enriched in protein processing in endoplasmic reticulum, protein export, and antigen processing and presentation, while genes in the Ep C3 subcluster were enriched in endocytosis, tight junction, and mitophagy.

Figure 2Heterogeneity of epithelial cells in cervical cancer based on the dataset GSE208653. (a) t‐SNE dimensionality reduction map for identifying the subclusters of epithelial cells. (b) The specifically highly expressed genes in the identified subclusters of epithelial cells. The *x*‐axis represents the identified subclusters of epithelial cells, and the *y*‐axis represents the specifically highly expressed genes. (c–e) KEGG functional enrichment analysis on the specifically highly expressed genes in each identified subcluster of epithelial cells. The *x*‐axis represents the number of size, and the *y*‐axis represents the name of the enriched item. The corresponding *p* value was denoted in different colors.

**Figure:**
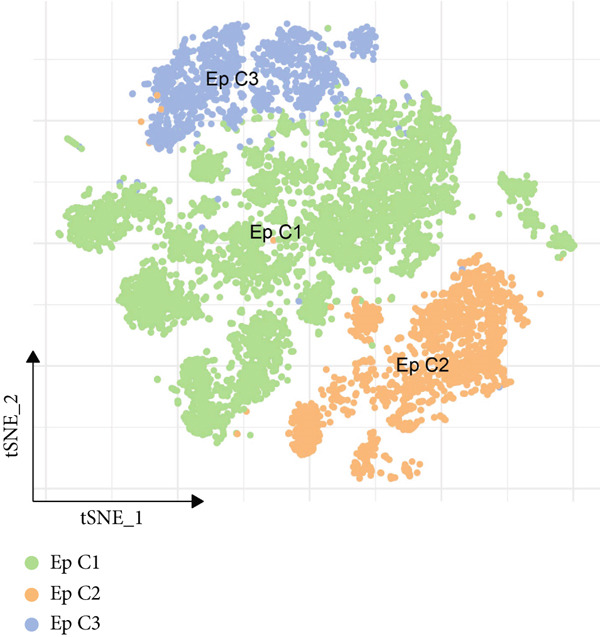
(a)

**Figure:**
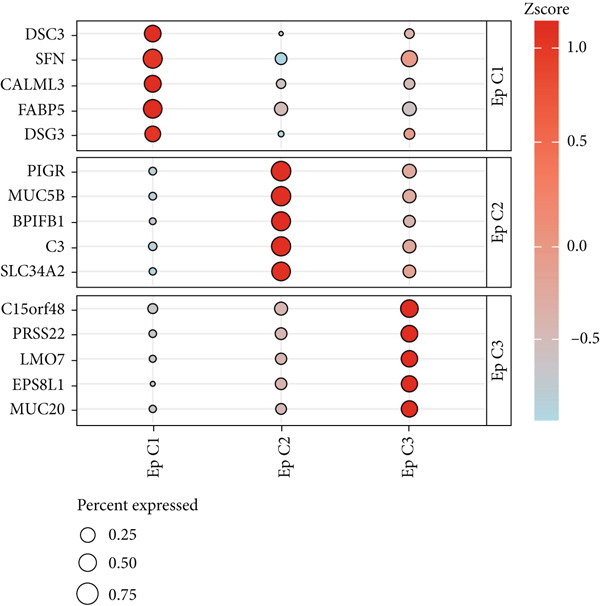
(b)

**Figure:**
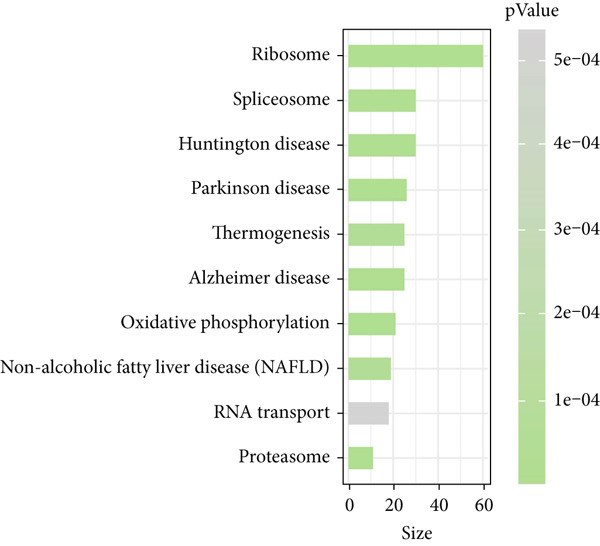
(c)

**Figure:**
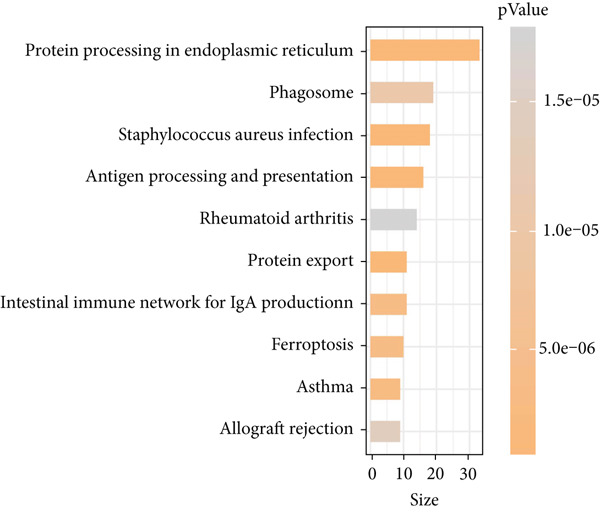
(d)

**Figure:**
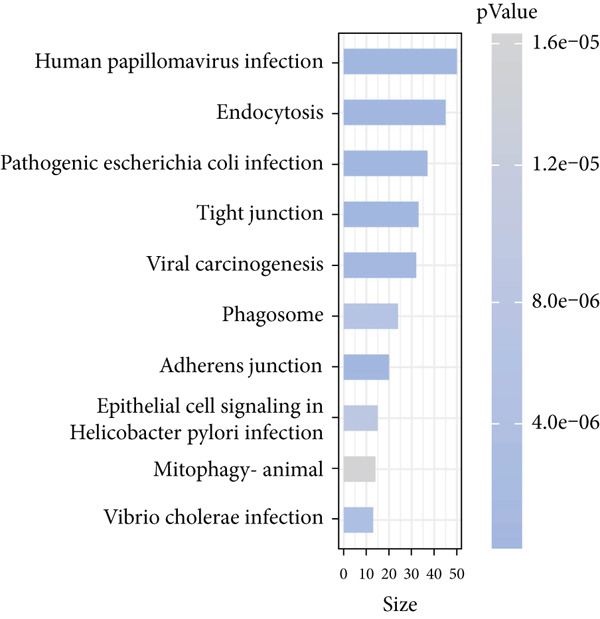
(e)

### 3.3. Pseudotime Analysis on the EpCs in CC

The monocle2 package was applied to plot the differentiation trajectory of the EpCs and for the pseudotime analysis. During the analysis, the cell population from normal samples with more branch points was taken as the starting point to mimic the progression of EpCs from normal status to cancerous status (Figure 3a). According to the results, EpCs may differentiate into two main trajectories of fate, based on which the genes differentially expressed based on the branch points were displayed in a heatmap (Figure 3b) and analyzed for the KEGG enrichment analysis. It was illustrated that genes in EpCs with the Differentiation Fate 1 (State 2 in the figure) were mainly enriched in Epstein–Barr virus infection, tight junction, antigen processing and presentation, and cell adhesion (Figure 3c), while those in EpCs with the Differentiation Fate 2 (State 3 in the figure) were enriched in ribosome, oxidative phosphorylation, and thermogenesis (Figure 3d). Hence, it could be assumed that EpCs may differentiate into two main fates associated with either immune defense or energy metabolism in CC.

Figure 3Pseudotime analysis on the epithelial cells in cervical cancer. (a) Differentiation trajectory of the epithelial cells from normal progression to cancerous differentiation and the differentiation fates. (b) Heatmap displaying the genes differentially expressed in epithelial cells during the different differentiation fates. (c, d) KEGG enrichment analysis on the genes differentially expressed based on the branch points. The *x*‐axis represents the gene ratio, and the *y*‐axis represents the name of enriched items. The shape of the bubble indicates the gene count, and the adjusted *p* value was shown in different colors.

**Figure:**
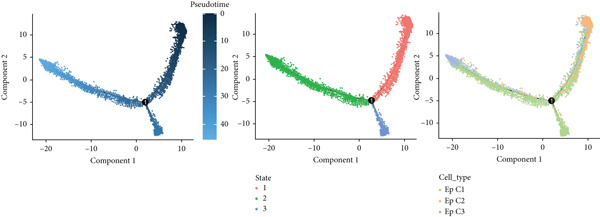
(a)

**Figure:**
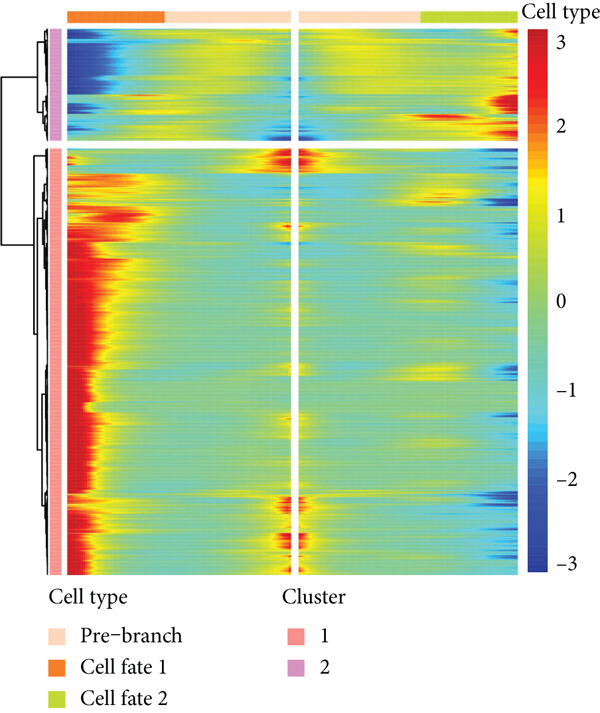
(b)

**Figure:**
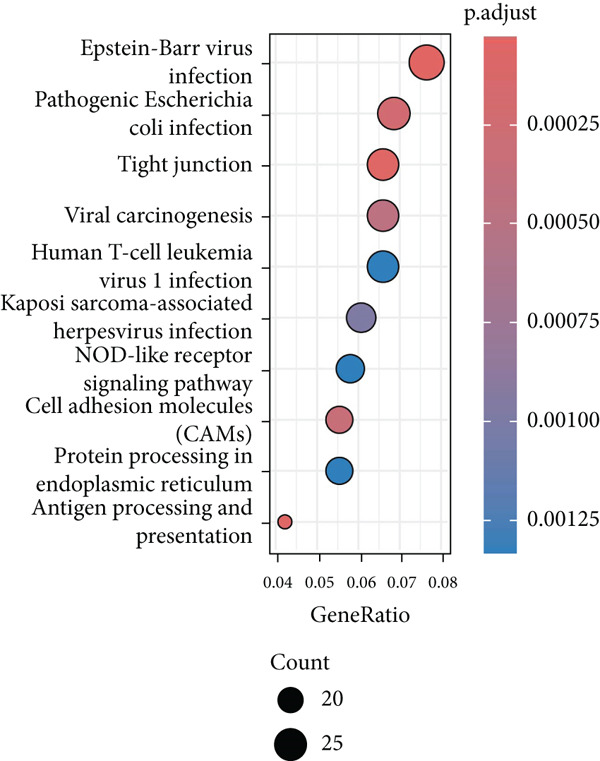
(c)

**Figure:**
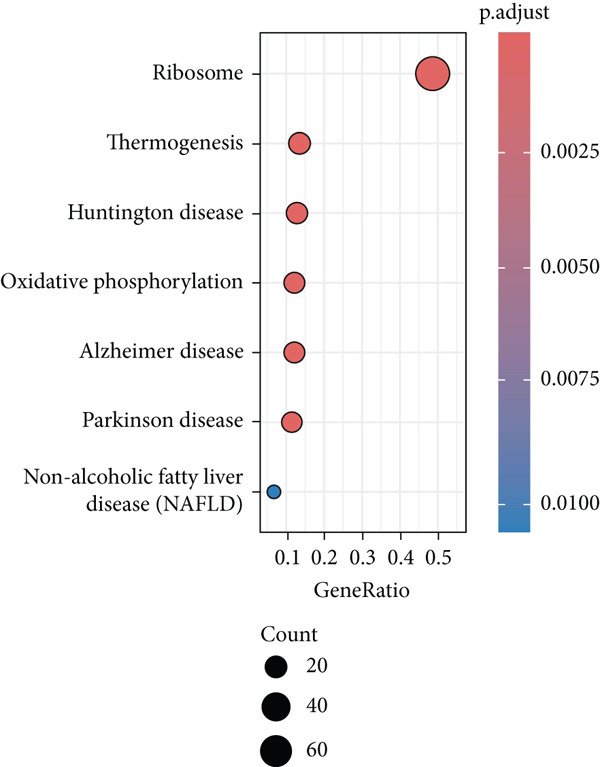
(d)

### 3.4. Cell–Cell Communication Analysis

The CellChat package was applied for the cell–cell communication analysis based on the CC and normal samples, and strong communication was seen in CC samples, as denoted by the increased number of inferred interactions (Figure 4a). The communication in the CC and normal samples was displayed in a circle plot, where a strong communication of EpCs with other cells in CC samples was also noticed (Figure 4b). Thereafter, with the purpose of exploring the involvement of EpCs in CC, the ssGSEA was applied to the highly expressed genes in EpCs based on the TCGA‐CESC cohort. The patients of the TCGA‐CESC cohort were then allocated to a high or low ssGSEA score group based on the optimal cutoff, and a worse prognosis was observed in high‐score patients (Figure 4c). Then, the ligand–receptor pairs underlying the mutual communication between other immune cells and Ep C3 of both CC and normal samples were displayed in the bubble plots of Figure 4d,e. The peculiar ligand–receptor pairs of LGALS9‐CD44, LGALS9‐CD45, and HBEGF‐EGFR were visible in the CC samples (Figure 4d,e).

Figure 4Cell–cell communication analysis based on epithelial cell Subcluster 3 and immune cells in cervical cancer. (a) Comparison of the number of inferred interactions in the cervical cancer sample (red) and normal sample (blue). (b) The number of interactions in the cervical cancer sample and normal sample, and the communication between the epithelial cell Subcluster 3 (signal source) and immune cells. (c) The overall survival of patients in the TCGA‐CESC cohort with high or low epithelial cell Subcluster 3 score. (d, e) The bubble plot unveils the mutual communication between epithelial cell Subcluster 3 and immune cells in CC and normal samples. The *x*‐axis represents the name of the interaction of epithelial cell Subcluster 3 and immune cells, and the *y*‐axis represents the predicted ligand–receptor pairs. The shape of the dots indicates the *p* value, and the communication probability is shown in different colors.

**Figure:**
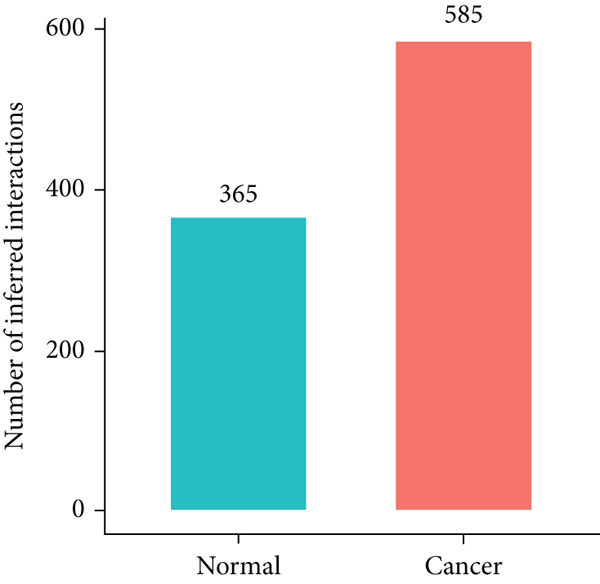
(a)

**Figure:**
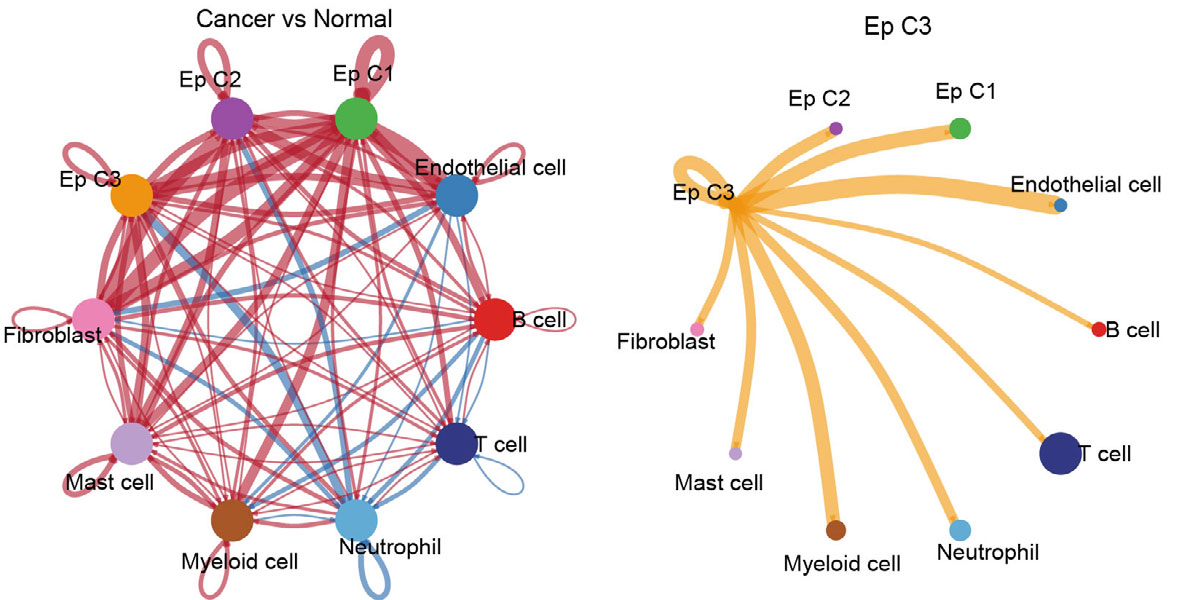
(b)

**Figure:**
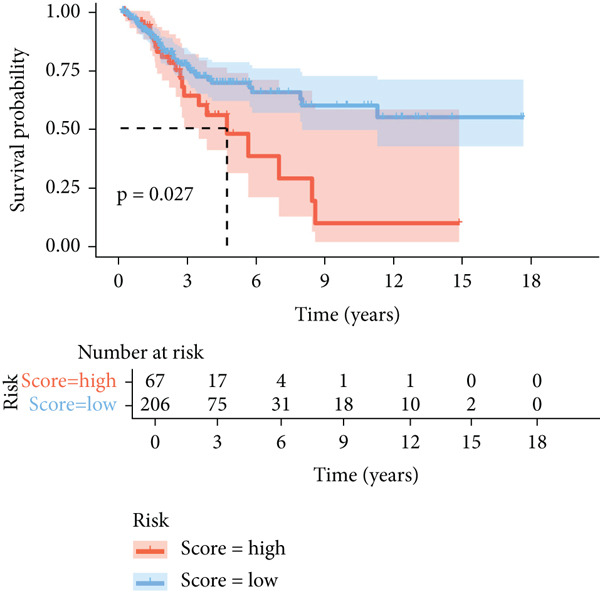
(c)

**Figure:**
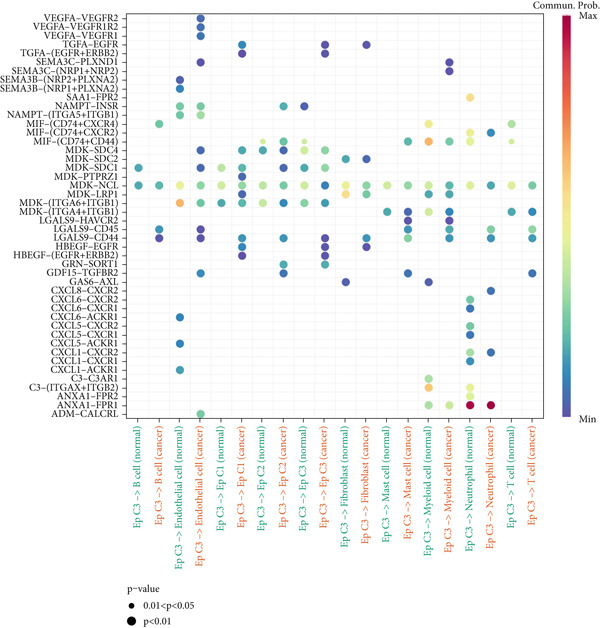
(d)

**Figure:**
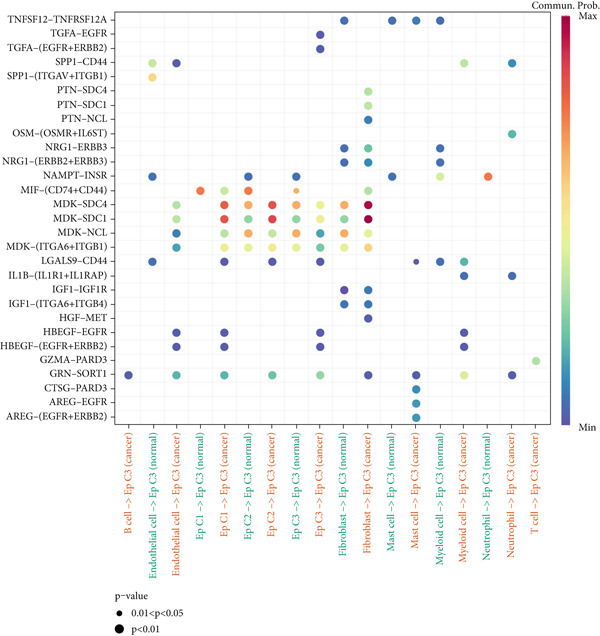
(e)

### 3.5. hdWGCNA Analysis on Identifying Ep C3–Related Gene Modules

Thereafter, the hdWGCNA analysis was initiated to reveal the specific gene modules to Ep C3, and the corresponding results on parameter sweep for the analysis are shown in Figure 5a. Based on the results, the soft threshold was set as 28, and the coexpression network was accordingly plotted (Figure 5b). The connectivity between the module trait value and the module was calculated thereafter, and the gene modules (*n* = 7) were determined based on the hub genes to each gene module (Figure 5c). The score of each module for each identified cell population was further calculated, and a relatively higher score was seen in the modules M1 and M5, respectively (Figure 5d). These two modules, accordingly, were applied for subsequent analysis.

Figure 5hdWGCNA analysis on identifying Ep C3–related gene modules. (a) Sorting procedures for the optimal soft threshold for hdWGCNA analysis with soft power threshold as the *x*‐axis and median or max connectivity as the *y*‐axis. (b) hdWGCNA dendrogram of Ep C3 to identify the relevant gene modules. (c) The first 10 feature genes of each identified gene module from the hdWGCNA analysis, ranked based on the kME value. (d) The activity of each gene module from the hdWGCNA analysis for each identified cell populations based on the percentage expressed (shown in dots) and the average expression (shown in different colors).

**Figure:**
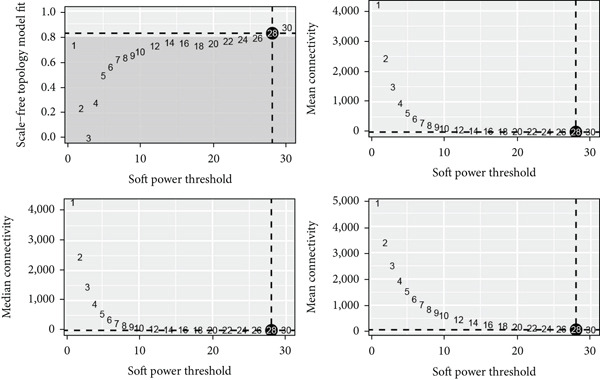
(a)

**Figure:**
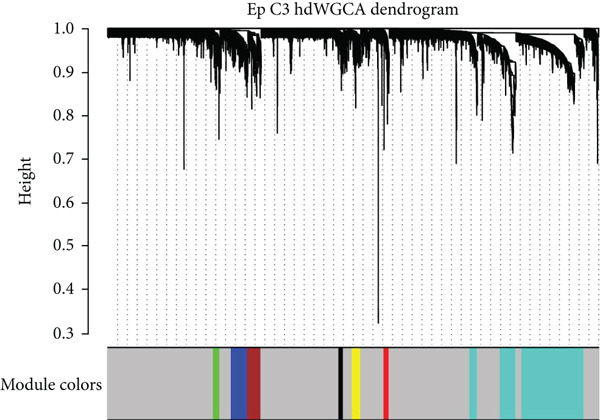
(b)

**Figure:**
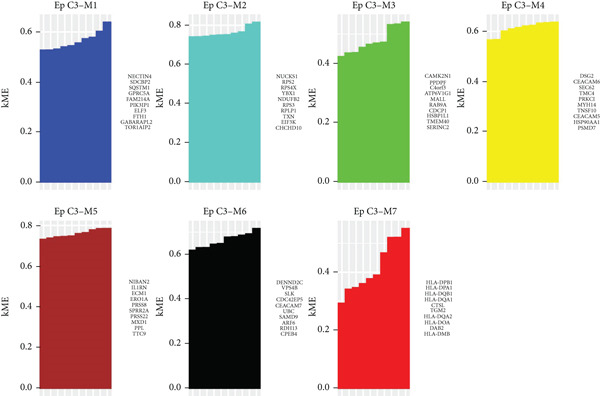
(c)

**Figure:**
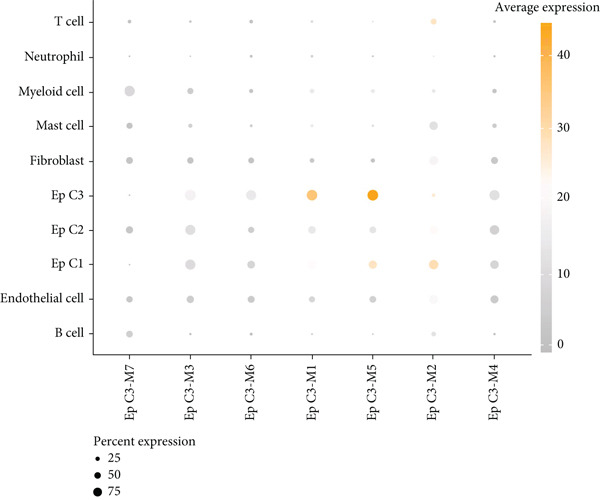
(d)

### 3.6. Construction of the Risk Model and Validation on Its Prognostic Efficacy

The Top 50 genes in the gene modules M1 and M5 from hdWGCNA analysis were intersected and taken as the module‐relevant genes, which were then subjected to the univariate Cox regression analysis to reveal the prognostically relevant genes (*n* = 19). The LASSO regression analysis was further performed on these 19 genes, and their trajectories with the change of the lambda value are shown in Figure 6a. Eleven genes were accordingly retained and applied for the multivariate Cox regression analysis, based on which five genes were finally recognized and applied for the risk model using the formula (Figure 6b):

Figure 6Construction of the risk model and validation on its prognostic efficacy. (a) The trajectories of each independent variable with the lambda and the confidence interval under each lambda. (b) Forest map displaying the key genes for the risk model. (c) The Riskscore on stratifying patients into high/low risk and the expression levels of the key genes in the cohort TCGA‐CESC. (d) The calculated AUC values of the risk model on predicting the 1‐ to 5‐year overall survival and the predicted overall survival of patients in high‐/low‐risk group of the cohort TCGA‐CESC. (e) The Riskscore on stratifying patients into high/low risk and the expression levels of the key genes in the cohort GSE52903. (f) The calculated AUC values of the risk model on predicting the 1‐ to 5‐year overall survival and the predicted overall survival of patients in high‐/low‐risk group of the cohort GSE52903.  ^∗∗^ *p* < 0.01 and  ^∗∗∗^ *p* < 0.001.

**Figure:**
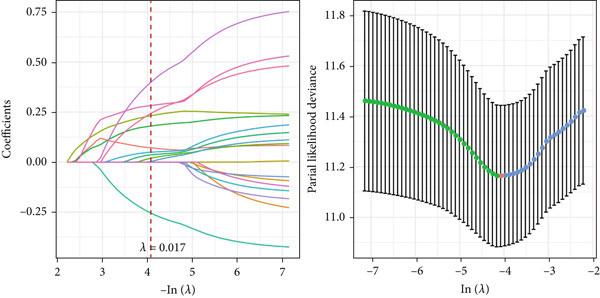
(a)

**Figure:**
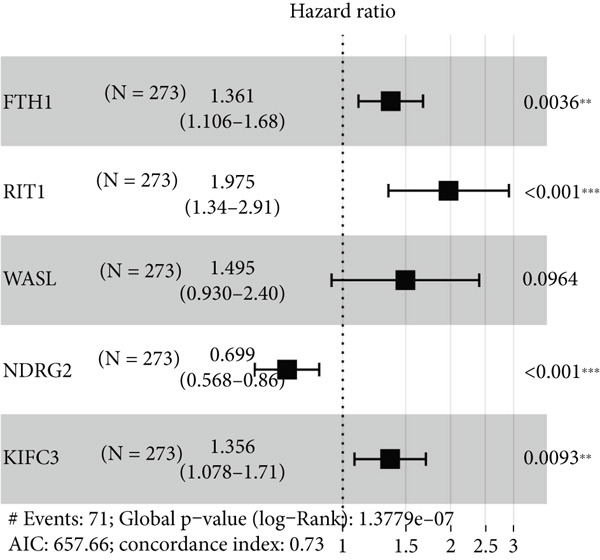
(b)

**Figure:**
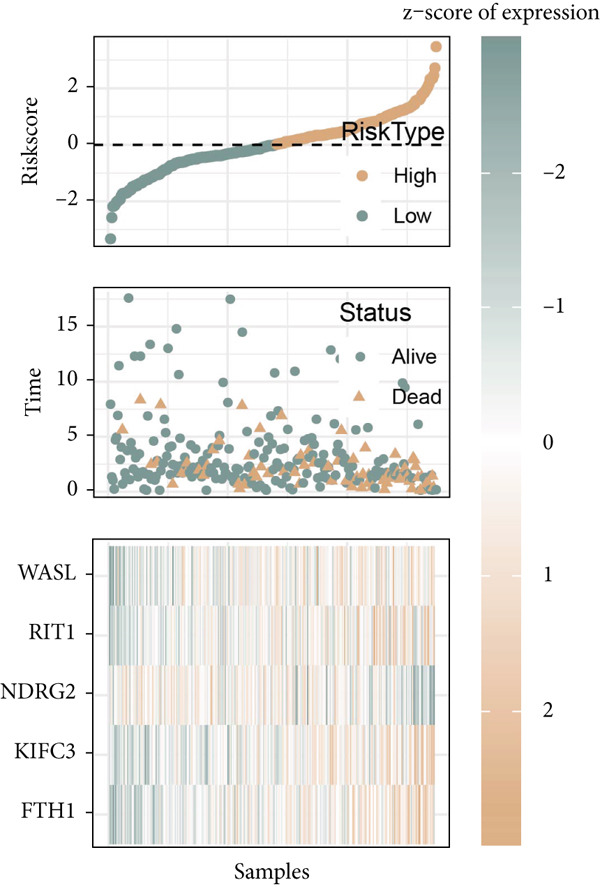
(c)

**Figure:**
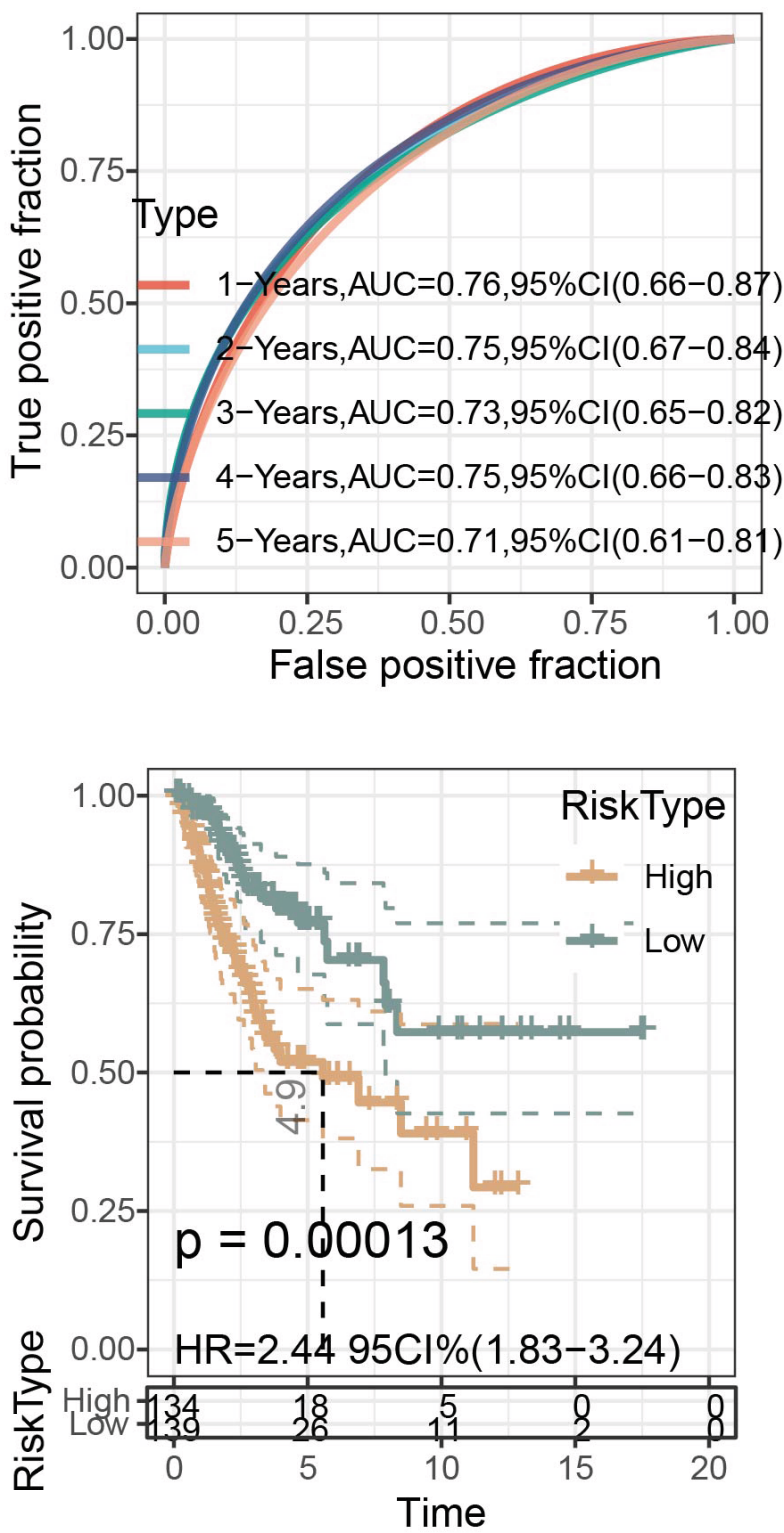
(d)

**Figure:**
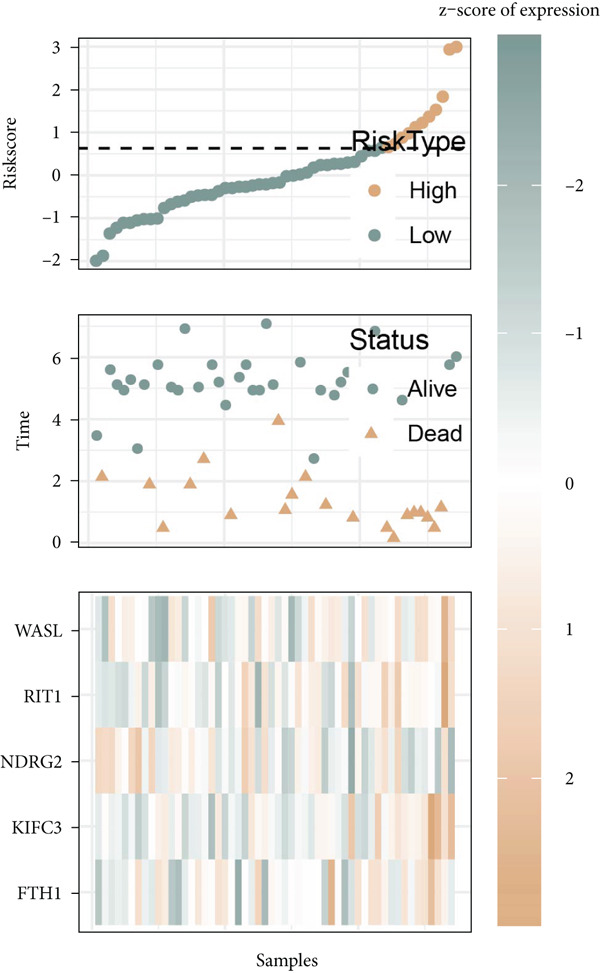
(e)

**Figure:**
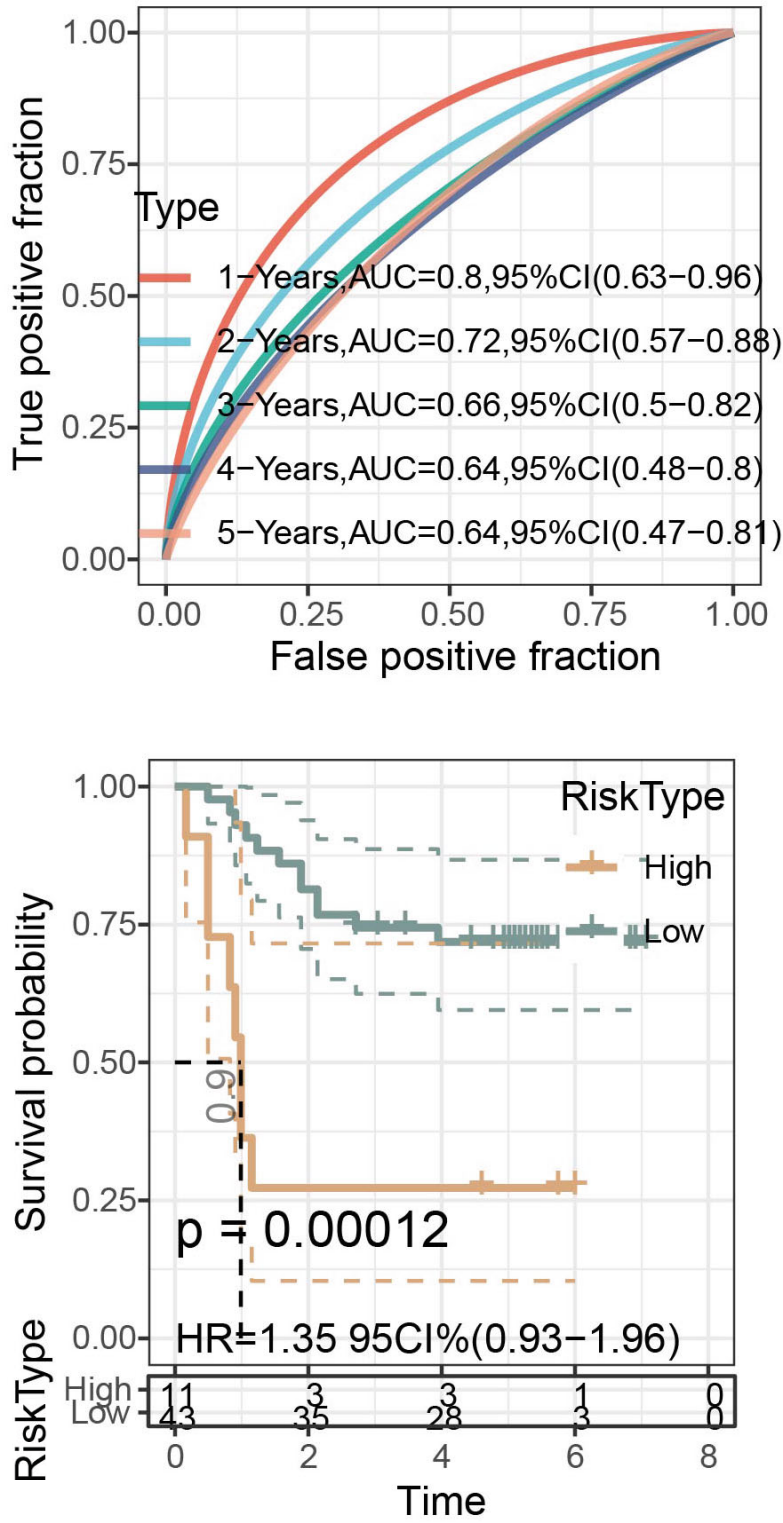
(f)

Riskscore = +0.308∗*F* *T* *H*1 + 0.681∗*R* *I* *T*1 + 0.402∗*W* *A* *S* *L* − 0.358∗*N* *D* *R* *G*2 + 0.304∗*K* *I* *F* *C*3.

The corresponding Riskscore was then applied to divide patients into either high‐risk (*n* = 134) or low‐risk (*n* = 139) group based on the median of the *z*‐score‐standardized Riskscore (Figure 6c), and the overall survival of patients in the two groups was compared. In accordance with the data from TCGA‐CESC, patients with a high Riskscore were related to a poorer overall survival, with the average AUC values of the Riskscore on predicting the overall survival > 0.7 (Figure 6d). Similarly, the dataset GSE52903 was applied to test the robustness of the risk model, and patients were also allocated to the high‐/low‐risk group based on the median value (Figure 6e). Similar results were also observed, with a poorer overall survival in high‐risk patients and an average AUC value on predicting the overall survival of > 0.7 (Figure 6f).

### 3.7. Determination of the Association of the Risk Model With TME and Chemotherapeutic Drug Sensitivity

The following scores, including ImmuneScore, StromalScore, and ESTIMATEScore, of patients in the high‐/low‐risk group were calculated using the ESTIMATE algorithm, and the lower scores were noticed in patients of high risk (Figure 7a). Also, the MCPCounter algorithm and ssGSEA were both applied to quantify the infiltration status of the immune cells of patients in the high‐/low‐risk group, unveiling a differential infiltration of the immune cells (Figure 7b,c). Further, the correlation between the Riskscore (as well as its key genes) and the IC_50_ values of chemotherapeutic drugs was determined, and most of the key genes were seen to be positively correlated with the IC_50_ values, while *NDRG2* was observed to be negatively correlated with the IC_50_ values (Figure 7d).

Figure 7Determination of the association of the risk model with immune infiltration and chemotherapeutic drug sensitivity. (a) Immune infiltration status in high‐/low‐risk patients of the TCGA‐CESC cohort analyzed via the ESTIMATE algorithm. (b, c) Scores of immune cells in high‐/low‐risk patients of the TCGA‐CESC cohort under the analysis of (b) ssGSEA and (c) MCPCounter algorithm. (d) The correlation analysis between the Riskscore (as well as its key genes) and the predicted IC_50_ of chemotherapeutic drugs.  ^∗^ *p* < 0.05,  ^∗∗^ *p* < 0.01, and  ^∗∗∗^ *p* < 0.001.

**Figure:**
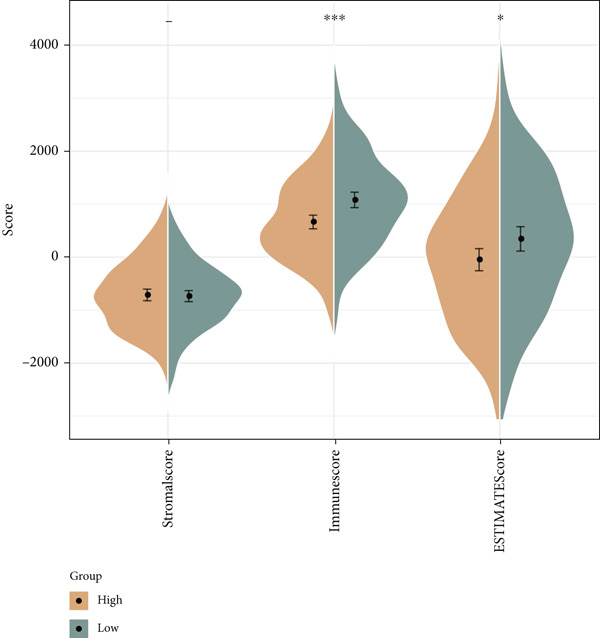
(a)

**Figure:**
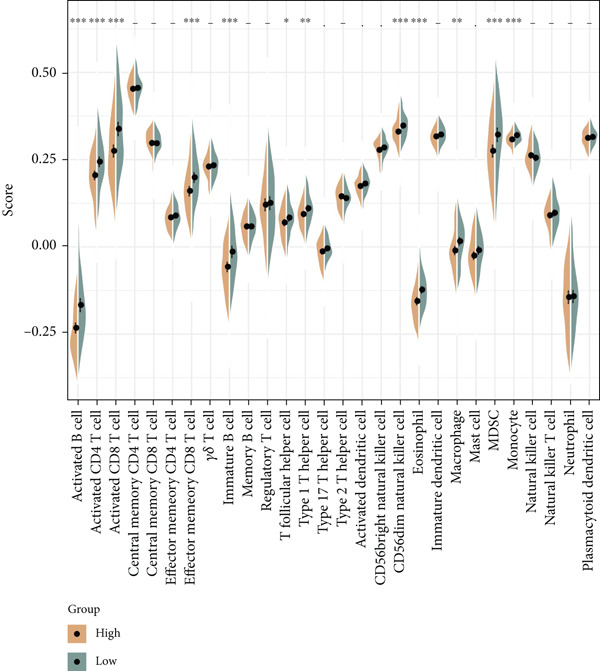
(b)

**Figure:**
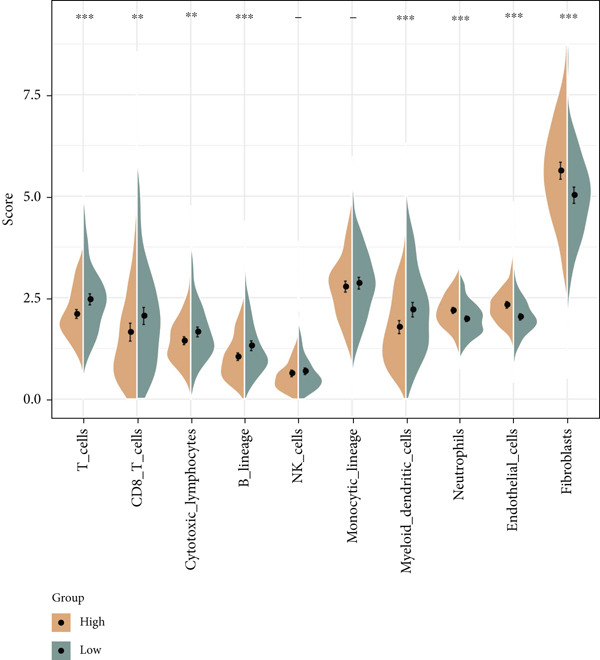
(c)

**Figure:**
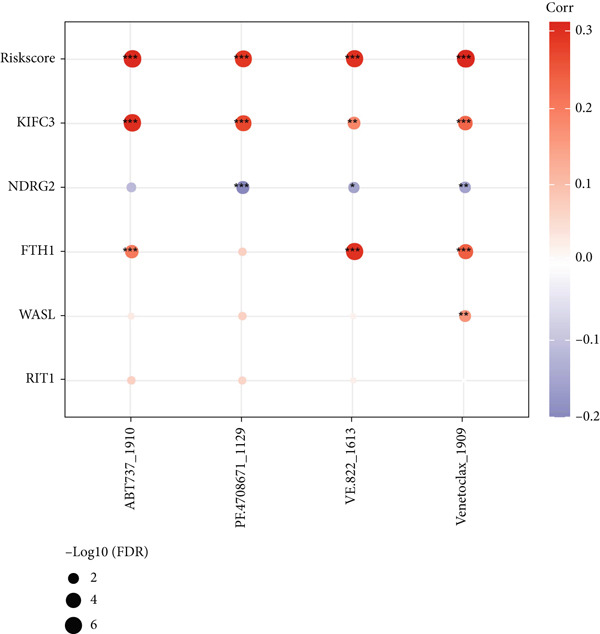
(d)

### 3.8. In Vitro Cell Assays to Evaluate the Expression and Potential Function of Key Genes

With the purpose of exploring the potential involvement of these five genes in CC, we firstly calculated their expression levels in CC cells and hEEC, and the expression levels of *FTH1*, *RIT1*, *WASL*, and *KIFC3* were higher, yet that of *NDRG2* was lower in CC cells HeLa and MS751 (Figure 8a). Based on previous studies, *FTH1*, functioning as a pivotal regulator of iron metabolism, has been established to promote tumor progression and confer chemoresistance in various malignancies by enhancing intracellular iron storage and suppressing ferroptosis [29, 30]. Furthermore, direct evidence exists demonstrating its role in promoting malignant phenotypes in CC [31], thereby justifying its selection for in‐depth functional investigation in our study. Toward this end, the siRNAs targeting *FTH1* were customized and transfected into CC cells HeLa and MS751, and the downregulated *FTH1* mRNA level in these CC cells hinted at the successful transfection (Figure 8b,c). Subsequently, we also observed that *FTH1* knockout resulted in a significant decrease in the proliferative capacity of HeLa and MS751 cells (Figure 8d,e), while markedly increasing their apoptotic potential (Figure 8f,g). Furthermore, *FTH1* knockout reduced the migration and invasion capabilities of CC cells (Figures 9a, 9b, 9c, and 9d). Collectively, these findings demonstrate that *FTH1* plays a critical oncogenic role in CC by promoting proliferation, inhibiting apoptosis, and enhancing migratory and invasive capabilities, highlighting its potential as a therapeutic target.

Figure 8Molecular validation on the implication of *FTH1* in CC cells in vitro. (a) The quantified mRNA levels of the five key genes of the risk model in cervical cancer cells HeLa and MS751 and human endometrial epithelial cells. (b, c) Validation on the knockdown efficiency of FTH1‐specific small interfering RNAs in cervical cancer cells (b) HeLa and (c) MS751. (d, e) CCK‐8 assay on evaluating the effects of FTH1 knockdown on the viability of cervical cancer cells (d) HeLa and (e) MS751. (f, g) Flow cytometry experiments were employed to investigate the effect of FTH1 knockdown on the in vitro apoptotic capacity of cervical cancer (f) HeLa and (g) MS751 cells. All experimental data were expressed as mean ± standard deviation.  ^∗^ *p* < 0.05,  ^∗∗^ *p* < 0.01,  ^∗∗∗^ *p* < 0.001, and  ^∗∗∗∗^ *p* < 0.0001.

**Figure:**
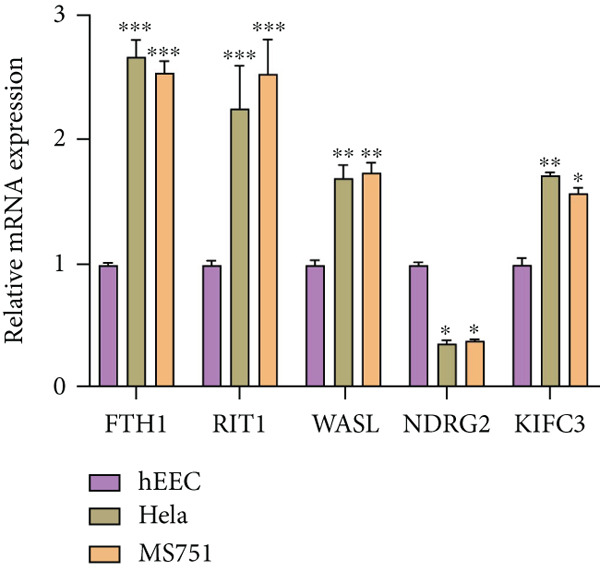
(a)

**Figure:**
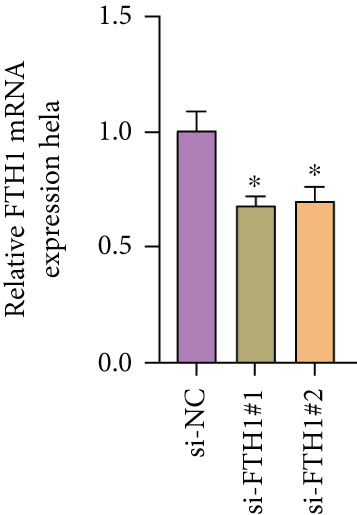
(b)

**Figure:**
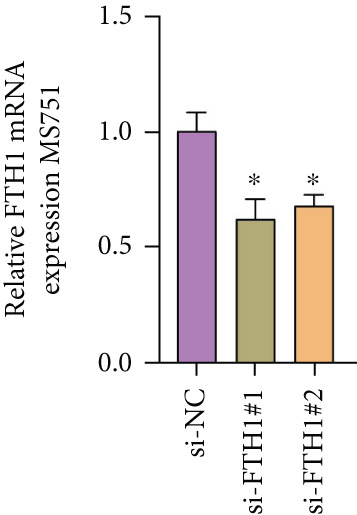
(c)

**Figure:**
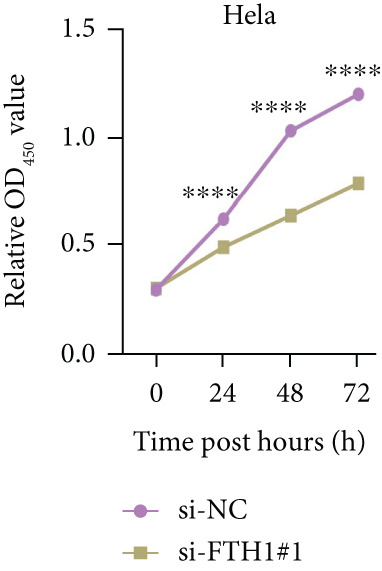
(d)

**Figure:**
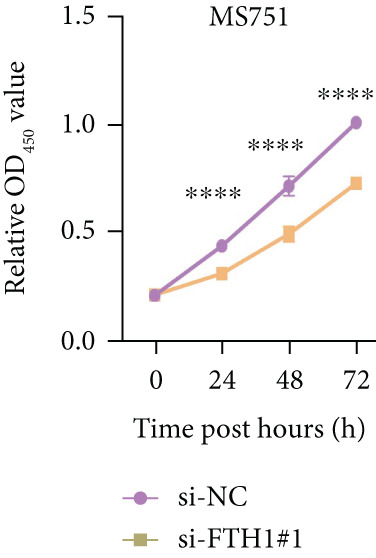
(e)

**Figure:**
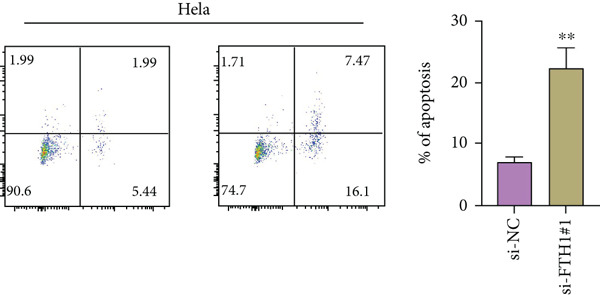
(f)

**Figure:**
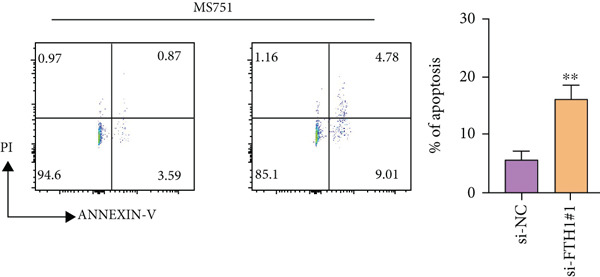
(g)

Figure 9Effects of *FTH1* knockout on migration and invasion capabilities of CC cells in vitro. (a) Scratch and (b) Transwell assays were applied to explore the effects of *FTH1* knockdown on the migration and invasion of CC cells HeLa in vitro. (c) Scratch and (d) Transwell assays were applied to explore the effects of *FTH1* knockdown on the migration and invasion of cervical cancer cells MS751 in vitro. All experimental data were expressed as mean ± standard deviation.  ^∗^ *p* < 0.05,  ^∗∗^ *p* < 0.01,  ^∗∗∗^ *p* < 0.001, and  ^∗∗∗∗^ *p* < 0.0001.

**Figure:**
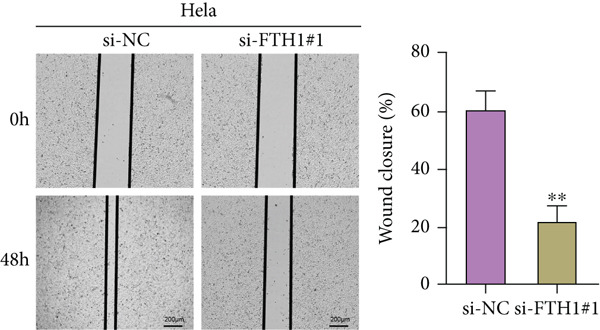
(a)

**Figure:**
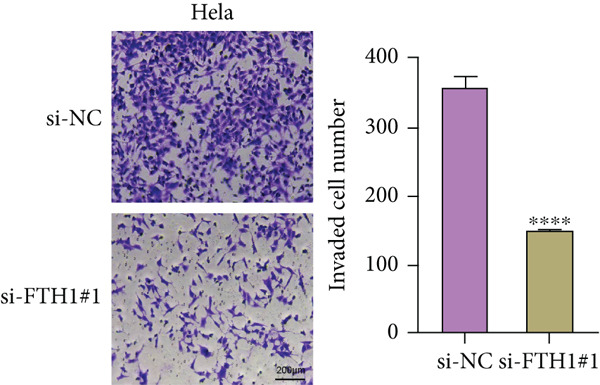
(b)

**Figure:**
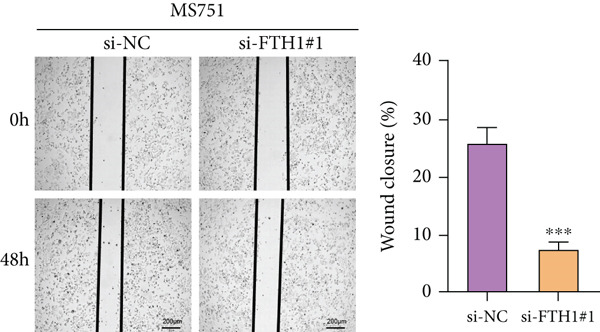
(c)

**Figure:**
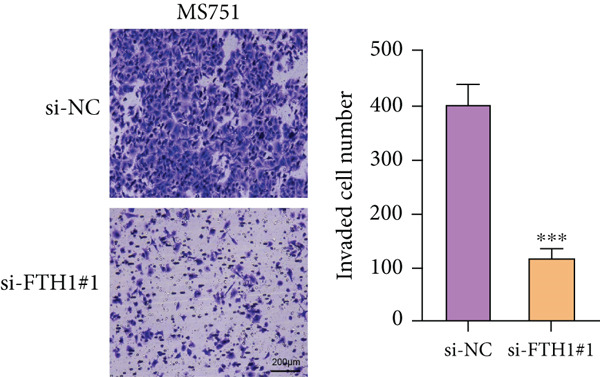
(d)

## 4. Discussion

In the present study, the scRNA‐seq data of HPV‐infected CC samples and normal samples from the dataset GSE208653 and the RNA‐seq data of CC samples from the datasets TCGA‐CESC and GSE52903 were combined to establish an EpC‐related prognostic model of CC which consists of a five‐gene signature and displays a good prognostic efficacy, thereby providing a reference for the molecular targets for personalized therapy in CC.

scRNA‐seq has profoundly advanced our understanding of cellular heterogeneity in CC, leading to prognostic models based on immune cells such as T cells and mast cells [32–34]. Although the Hedgehog pathway has been implicated in EpC dynamics during CC progression [35], a comprehensive dissection of EpC heterogeneity and its clinical relevance remains limited. Here, leveraging the GSE208653 dataset, we identified three distinct EpC subclusters with unique functional identities. Ep C1 was enriched in metabolic and biosynthetic processes including ribosome and oxidative phosphorylation [36], whereas Ep C2 displayed strong involvement in antigen processing and presentation—a key determinant of adaptive antitumor immunity [37]. Notably, Ep C3, which demonstrated active communication with immune cells, was characterized by pathways regulating cell adhesion, polarity, and microenvironment interaction, such as tight junction, endocytosis, and mitophagy [38–40]. Pseudotime trajectory analysis further revealed two divergent differentiation fates: one oriented toward immune and barrier functions and the other toward enhanced metabolism and protein synthesis. These findings underscore the functional diversity of EpCs in CC and highlight Ep C3 as a key mediator of tumor immune crosstalk.

Cell–cell communication has been underlined as an essential mechanism driving the maintenance and development of multiple organs, including the female reproductive system [41]. In the context of CC, the communication between EpCs and macrophages has been documented, which may be realized via the SPP1‐CD44 axis [42]. Another study has underlined that the neoadjuvant chemotherapy has led to decreased interaction strength between T cells and cancer cells, yet intensified interaction strength between macrophages and cancer cells [43]. Further analysis on CSCC and CAde has suggested that the pairs of NRG1‐ERBB2 and FN1‐ITGA3 may be specific to CSCC and CAde [44]. In the present study, which mainly focused on the communication between Ep C3 and other immune cells in CC, the following pairs, including LGALS9‐CD44, LGALS9‐CD45, and HBEGF‐EGFR, were identified. While the involvement of such ligand–receptor pairs LGALS9‐CD44 and LGALS9‐CD45 in CC awaits further research, prior evidence has demonstrated that these two pairs may be two pivotal interactions between blast cells and regulatory T cells in the research of acute myeloid leukemia [42]. In the meantime, elevated HBEGF has been seen in both the epithelium and stroma of CC, while EGFR has been extensively discussed as a biomarker for the prognosis or the treatment of CC [43, 45]. Moreover, HBEGF‐EGFR has been suggested as a key mediator underlying the crosstalk between cancer cells and cancer‐associated fibroblasts in CC [43]. These discoveries, collectively, provided some novel clues on the molecular mechanisms related to the development and progression of CC.

Thereafter, the Ep C3 was further applied as the subcluster of EpCs to reveal some feature genes using hdWGCNA analysis, which were additionally narrowed down to obtain the key genes for our Riskscore model. A 13 necroptosis‐related gene prognostic signature was developed and validated in the TCGA‐CESC cohort, with an average AUC > 0.7 in the 3‐, 5‐, and 10‐year ROC curve [46]. Another five‐hypoxia‐related gene signature was also developed, and the results from the TCGA‐CESC (training set) and the GSE44001 (validation set) have demonstrated a good risk prediction effect [47]. In our current study, only five genes (*FTH1*, *RIT1*, *WASL*, *NDRG2*, and *KIFC3*) were selected for the Riskscore model. While the specific association between *FTH1*, *WASL*, *NDRG2*, and *KIFC3* and the Riskscore awaits further elaboration, *RIT1* has been already characterized as an Nrf2 signaling pathway‐related gene which could be applied to establish a prognosis model significantly associated with the survival and the clinicopathological characteristics in lung squamous carcinoma, breast cancer, and gastric cancer [48]. Moreover, several published studies have manifested that *FTH1* mRNA stability may be compromised via *METTL14* through N6‐methyladenosine (m6A) modification to enhance sorafenib‐induced ferroptosis in CC and that *NDRG2* knockdown could sensitize CC cells HeLa to cisplatin via repressing Bcl‐2 expression [31, 49]. In accordance with the results of the present study, the aforementioned five genes were taken for the construction of the Riskscore model, and the cohorts of TCGA‐CESC and GSE52903 were adopted to validate the efficacy. Based on the data from TCGA‐CESC, patients with a high Riskscore were related to a poorer overall survival, with the average AUC values of the Riskscore on predicting the 1‐ to 5‐year overall survival > 0.7. Similarly, in the dataset GSE52903, an average AUC value of the Riskscore on predicting the overall survival was also > 0.7. These results have therefore hinted at the robustness of our EpC‐related gene signature in predicting the overall survival of CC patients, thereby providing some ideas for clinical decision‐making.

The crucial role of tumor immune microenvironment in CC has been further emphasized [50]. A previous study focusing on the RNA‐seq from the TCGA‐CESC cohort has displayed a bar chart of 22‐type immune cell proportion along with the differential score of StromalScore, ImmuneScore, and ESTIMATEScore in HPV^+^ and HPV^−^ CC patients [32]. Further, a low and recently activated TME was noticed in high‐stage intraepithelial neoplasia, which was characterized by a high infiltration of tissue‐resident CD8 T cell, effector NK cells, Treg, DC1, pDC, and M1‐like macrophages, while an immunosuppressive TME was observed in tumor tissue, as exemplified by the enrichment of exhausted CD8 T cells, resident NK cells, and M2‐like macrophages [51]. In our present study, in addition to a lower score of StromalScore, ImmuneScore, and ESTIMATEScore in CC patients of high risk, a differentially enriched score of tumor‐infiltrating immune cells was also seen. Further, a negative correlation was noticed in the Riskscore with the IC_50_ values of certain drugs like ABT737, PF.4708671, VE.822, and venetoclax. Noteworthily, ABT737 has been identified as one of the candidate therapeutic drugs for ovarian cancer patients with a high metabolism‐related gene prognostic index score, while PF.4708671 has been recognized as a potential alternative drug for patients who develop resistance to anti‐PD‐1/PD‐L1 therapy [52, 53]. Also, VE.822 is listed as a potential drug for the treatment based on a tertiary lymphoid structure‐related gene signature in colon adenocarcinoma [54]. Further, despite extensive investigation on the efficacy in acute myeloid leukemia, venetoclax has been further characterized as a potential drug for pancreatic cancer in a recent research where a prognostic model was established using mitochondrial metabolism–related genes [55, 56]. These results, we hope, may be beneficial for the development of relevant personalized therapy in CC.

Some shortcomings in this study, nonetheless, should be addressed. First, the prognostic predictive performance and clinical relevance of the risk model have been validated only in retrospective public datasets from TCGA and GEO. Subsequent studies will collect prospective clinical cohorts encompassing diverse geographic regions and ethnic populations to conduct broader external validation of this risk model, thereby advancing its translation into clinical application. Second, although the carcinogenic function of *FTH1* has been demonstrated through in vitro experiments, the biological functions of other key genes in the model (such as *RIT1* and *WASL*) remain unvalidated. Moving forward, we will employ a comprehensive approach integrating gene editing, organoid models, and animal experiments to systematically elucidate the functions of all key genes in the model. This will enable us to delve deeper into the specific signaling pathways through which core genes like *FTH1* regulate the progression of CC. Finally, while this study has revealed significant associations between risk scores and the immune microenvironment as well as drug sensitivity through bioinformatics methods, these computational predictions lack empirical support from in vivo experiments or clinical samples. Therefore, we plan to validate these findings through spatial transcriptomics, multiomics analysis, and clinical patient tissue samples using multiplex immunofluorescence. This will establish the intrinsic connection between risk scores and tumor immune landscapes at the molecular and pathological levels. Furthermore, we will conduct preclinical efficacy assessments using patient‐derived xenograft models to provide a robust foundation for personalized treatment strategies.

## 5. Conclusion

In conclusion, our integrated analysis of single‐cell and bulk transcriptomic data reveals significant EpC heterogeneity in CC. We developed a novel five‐gene prognostic signature derived from EpC‐specific gene modules that demonstrates robust performance in risk stratification across multiple validation cohorts. The risk score further correlates with distinct tumor immune microenvironment profiles and chemotherapeutic sensitivity patterns. Experimental validation established *FTH1*′s crucial role in promoting CC cell proliferation and invasion. These findings provide both a reliable prognostic tool and valuable insights into the tumor biology of CC, supporting future research into EpC‐specific therapeutic strategies.

**Nomenclature**
CC: cervical cancer
HPV: human papillomavirus
scRNA‐seq: single‐cell RNA sequencing
TME: tumor microenvironment
EpCs: epithelial cells
RNA‐seq: RNA sequencing
hdWGCNA: high‐dimensional weighted gene coexpression network
GEO: Gene Expression Omnibus
PCA: principal component analysis
UMAP: uniform manifold approximation and reduction
ROC: receiver operator characteristic
AUC: area under the curve
ssGSEA: single‐sample gene set enrichment analysis
IC_50_
: half‐maximal inhibitory concentration
hEEC: human endometrial epithelial cell
DMEM: Dulbecco′s modified Eagle′s medium
FBS: fetal bovine serum
cDNA: complementary DNA
CCK‐8: cell counting kit‐8
OD_450_
: optical density at 450 nm
CSCC: cervical squamous cell carcinoma
CAde: cervical adenocarcinoma
m6A: N6‐methyladenosine

## Ethics Statement

The authors have nothing to report.

## Disclosure

All authors provided the final approval of this manuscript.

## Conflicts of Interest

The authors declare no conflicts of interest.

## Author Contributions

Conception and design: Chengli Dou, Xiang Li, and Xiaojing Wang; administrative support: Biao Din; provision of study materials or patients: Xuegu Wang; collection and assembly of data: Zhixin Jin and Chengli Dou; data analysis and interpretation: Xiang Li, Xingchen Pan, and Zhixin Jin; manuscript writing: Chengli Dou and Xiaojing Wang.

## Funding

This work was funded by the Natural Science Key Program of Bengbu Medical University (2023byzd053) and the Key Program of Natural Science Research of Higher Education of Anhui Province (2024AH051291, 2023AH051992).

## Supporting information


**Supporting Information** Additional supporting information can be found online in the Supporting Information section. Figure S1: Results on the single‐cell data based on the dataset GSE208653 following quality control. (A–C) The corresponding (A) nFeature_RNA, (B) nCount_RNA, and (C) percent.mt of two normal samples (GSM6360680 and GSM6360681) and three HPV‐positive samples (GSM6360686, GSM6360687, and GSM6360688) based on the dataset GSE208653. Table S1: Target sequence (5 ^′^‐3 ^′^) for the transfection via liposome. Table S2: Primer sequences for PCR quantification assay.

## Data Availability

The data that support the findings of this study are available from the corresponding authors upon reasonable request.
