# Supplementary material for: Epithelial Cell–Specific Prognostic Signature (FTH1, RIT1, WASL, NDRG2, KIFC3) Stratifies Cervical Cancer Patients and Correlates With Immune Infiltration
Source: Hum Mutat. 2026 Feb 6;2026:4109928. doi: 10.1155/humu/4109928 (PMC12881713; doi:10.1155/humu/4109928)
Supplement: Supplementary file 1 — Supporting Information Additional supporting information can be found online in the Supporting Information section. Figure S1: Results on the single‐cell data based on the dataset GSE208653 following quality control. (A–C) The corresponding (A) nFeature_RNA, (B) nCount_RNA, and (C) percent.mt of two normal samples (GSM6360680 and GSM6360681) and three HPV‐positive samples (GSM6360686, GSM6360687, and GSM6360688) based on the dataset GSE208653. Table S1: Target sequence (5 ′‐3 ′) for the transfection via liposome. Table S2: Primer sequences for PCR quantification assay. [file HUMU-2026-4109928-s001.zip › Supplementary tables.docx]

**Table S1. Target sequence (5’-3’) for the transfection via liposome**

| Target | Purpose | Sequence (5’-3’) |
| --- | --- | --- |
| si-FTH1#1 | FTH1 knockdown | GCCTCGGGCTAATTTCCCATA |
| si-FTH1#2 | FTH1 knockdown | GCTTTGAAGAACTTTGCCAAA |
| si-NC | Negative control | CCTAATTCGGTATGACGCCTC |

**Table S2. Primers sequences for PCR quantification assay**

| Gene | NCBI  Accession No | Primers (5’->3’) | |
| --- | --- | --- | --- |
|  |  | Forward | Reverse |
| FTH1 | NM_002032.3 | AGAACTACCACCAGGACTC | GCTTGTCAAAGAGATATTCC |
| RIT1 | NM_001256821.2 | ACAGCTTATTTATCGAGTCC | TTTCTTTCCTACGTATCTCC |
| WASL | NM_003941.4 | GAAAAGCAGTTACAGACCTT | TTTTAGTTGGATACCCTGTC |
| NDRG2 | NM_201535.2 | ATATCAGTACCCATCTCTGG | GGTCCAGTTTTGAGTTACAT |
| KIFC3 | XM_006721188.2 | CTTTTCAGGAAGAACACTCT | CCCACCTGTATTCTCTACAT |
| GAPDH | NM_002046.7 | CTTCATTGACCTCAACTACA | AGGCTGTTGTCATACTTCTC |
