# Supplementary figures and images for: Epithelial Cell–Specific Prognostic Signature (FTH1, RIT1, WASL, NDRG2, KIFC3) Stratifies Cervical Cancer Patients and Correlates With Immune Infiltration
Source: Hum Mutat. 2026 Feb 6;2026:4109928. doi: 10.1155/humu/4109928 (PMC12881713; doi:10.1155/humu/4109928)

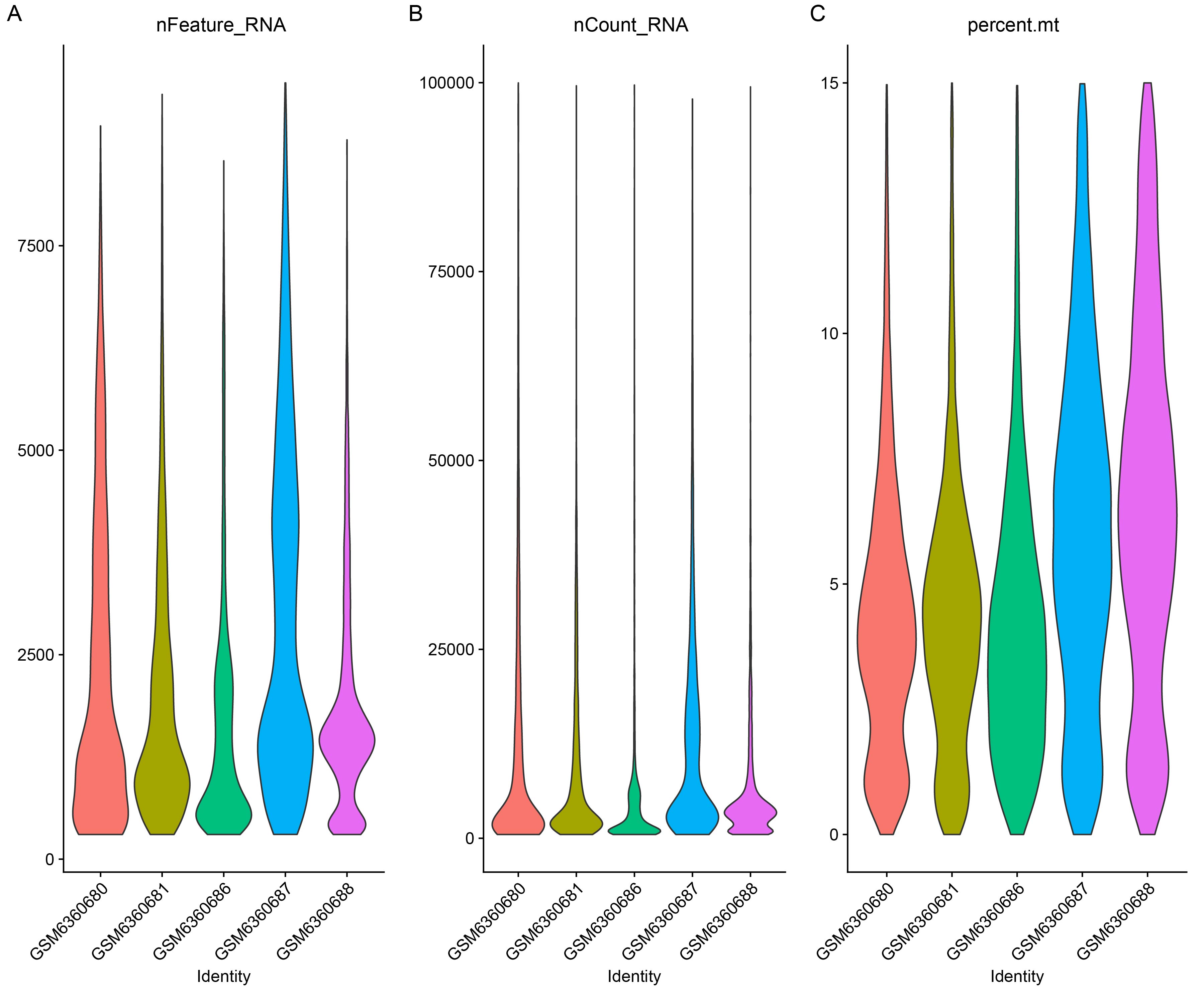

Supplement: Supplementary file 1 — Supporting Information Additional supporting information can be found online in the Supporting Information section. Figure S1: Results on the single‐cell data based on the dataset GSE208653 following quality control. (A–C) The corresponding (A) nFeature_RNA, (B) nCount_RNA, and (C) percent.mt of two normal samples (GSM6360680 and GSM6360681) and three HPV‐positive samples (GSM6360686, GSM6360687, and GSM6360688) based on the dataset GSE208653. Table S1: Target sequence (5 ′‐3 ′) for the transfection via liposome. Table S2: Primer sequences for PCR quantification assay. [file HUMU-2026-4109928-s001.zip › Figure S1.jpg]
